# The Final Size of a Serious Epidemic

**DOI:** 10.1007/s11538-018-00549-x

**Published:** 2018-12-07

**Authors:** Fred Brauer

**Affiliations:** Department of Mathematics, University of British Columbia, Vancouver, BC Canada

**Keywords:** Epidemic models, Final size relations, Behavioral response

## Abstract

In an epidemic of a serious disease, there is likely to be behavioral response that decreases the epidemic size considerably, and taking this into account may lead to estimates of the final epidemic size that are much smaller and more realistic than estimates that do not take this into account.

## Introduction

It has been standard practice in analyzing disease outbreaks to formulate a dynamical system as a deterministic compartmental model, then to use observed early outbreak data to fit parameters to the model, and finally to analyze the dynamical system to predict the course of the disease outbreak and to compare the effects of different management strategies. In general, such models predict an initial stochastic stage (while the number of infectious individuals is small), followed by a period of exponential growth. Measurement of this early exponential growth rate is an essential step in estimating contact rate parameters for the model. A thorough description of the analysis of compartmental models may be found in Hethcote (2000).

However, instances have been noted where the growth rate of an epidemic is clearly slower than exponential, especially for diseases which are viewed as very serious. For example, the initial apparently exponential spread of the 2013–2015 Ebola epidemic in West Africa can be viewed as a composition of locally asynchronous outbreaks at local level displaying sub-exponential growth patterns during several generations (Chowell et al. 2015). One of the earliest examples (Colgate et al. 1989) concerns the growth of HIV/AIDS in the USA, and a possible explanation might be the mixture of short-term and long-term contacts. This could be a factor in other diseases where there are repeated contacts in family groups and less frequent contacts outside the home. There may be more than one mechanism influencing the early epidemic growth rate, and it may not be possible to estimate the effects of different mechanisms from incidence data.

A variety of epidemiological situations in which slower than exponential epidemic growth might be possible have been described (Chowell et al. 2016b, a). Ultimately, the challenge for epidemiological modeling would be to determine which of these situations allow slower than exponential growth by deriving and analyzing mechanistic models to describe each of these situations. This is an important new direction for epidemic modeling. Some suggestions include metapopulation models with spatial structure including cross-coupling and mobility, clustering in spatial structure, dynamic contacts, agent-based models with differences in infectivity and susceptibility of individuals, and reactive behavioral changes early in a disease outbreak (Chowell et al. 2016a). It may well turn out that slower than exponential growth may be ruled out in some cases but is possible in others. For example, heterogeneity of mixing in a single location can be modeled by an autonomous dynamical system and the linearization theory of dynamical systems at an equilibrium shows that early epidemic growth for such a system is always exponential. On the other hand, metapopulation models may well allow many varieties of behaviors. It is likely that more than one mechanism is at work in decreasing the growth rate, and this would complicate disentangling the mechanisms involved form the incidence data.

There have been several approaches to the question of modeling disease outbreaks which grow more slowly than might be expected. It has been pointed out (Chowell et al. 2016a, b, 2015; Viboud et al. 2016) that a so-called general growth model of the form

$$\begin{aligned} C'(t) = r C(t)^p, \end{aligned}$$

where *C*(*t*) is the number of disease cases occurring up to time *t*, and $p, 0 \le p \le 1,$ is a “deceleration of growth” parameter, has exponential solutions if $p = 1$ but solutions with polynomial growth if $0< p < 1$. This is not and does not claim to be a mechanistic epidemic model, but it has proved to be remarkably successful for fitting epidemic growth and predicting the future course of an epidemic (Pell et al. 2018; Shanafelt et al. 2018). For example, it has provided much better estimates of epidemic final size than the exponential growth assumption for the Ebola epidemic of 2014. Such phenomenological models are particularly likely to be suitable in situations where it is difficult to construct a mechanistic approach because of multiple transmission routes, interactions of spatial influences, or other aspects of uncertainty.

We suggest that an assumption of a contact rate that decreases in time, because of behavioral response to an outbreak of a disease that is often fatal, is a very plausible assumption. For example, during the SARS epidemic of 2002–2003 many people began to wear face masks to try to prevent disease spread through contact with airborne particles, even though this may have in fact been counter-productive.

Another example of a behavioral response is given by the Great Plague in the village of Eyam near Sheffield, England, which suffered an outbreak of bubonic plague in 1665–1666, the source of which is generally believed to be the Great Plague of London (Raggett 1982). The Eyam plague was survived by only 83 of an initial population of 350 persons.

In Eyam, the rector persuaded the entire community to quarantine itself to prevent the spread of disease to other communities. He assumed that infection was transmitted directly between people. While this is possible, it was thought that bubonic plague is transmitted mainly by rat fleas. When an infected rat is bitten by a flea, the flea becomes extremely hungry and bites the host rat repeatedly, spreading the infection in the rat. When the host rat dies, its fleas move on to other rats, spreading the disease further. As the number of available rats decreases, the fleas move to human hosts, and this is how plague starts in a human population. It is believed now that fleas were the main agents of the spread of plague (Dean et al. 2018).

One effect of this policy was to increase the infection rate in the village by keeping fleas, rats, and people in close contact with one another, and the mortality rate from bubonic plague was much higher in Eyam than in London. Further, the quarantine could do nothing to prevent the travel of rats and thus did little to prevent the spread of disease to other communities.

## A Variable-Contact-Rate Epidemic Model

Another direction that would be well worth further exploration would be contact rates decreasing in time because of individual behavioral changes in response to a disease outbreak. A contact rate which is a decreasing function of time can certainly lead to early epidemic growth slower than exponential. A step in this direction has been initiated in a discrete model (Fisman et al. 2013) that has been applied to an Ebola model in Fisman et al. (2014).

Our goal is to develop a simple approach to the question of making estimates early in an outbreak of a dangerous disease for the eventual size of the epidemic, considering the total size of the epidemic over a country rather than the more complicated growth in separate villages. Study of the local structure of an epidemic is essential for attempts to control the epidemic; our intent is only to obtain early estimates of the full extent of an epidemic in order to judge the resources necessary for management.

The idea of a contact rate decreasing exponentially in time has been suggested in Chowell et al. (2004). In Althaus (2014), a model for Ebola has been described consisting of a standard *SEIR* epidemic model with disease deaths but with a contact rate which decreases exponentially in time,

1 $$\begin{aligned} S'= & {} - \beta e^{- \kappa t}S \frac{I}{P} \nonumber \\ E'= & {} \beta e^{- \kappa t}S \frac{I}{P} - \eta E \nonumber \\ I'= & {} \eta E - \gamma I \nonumber \\ R'= & {} (1 - f)\gamma I . \end{aligned}$$

Here, $\beta$ represents the transmission rate in unit time, depending on the mean number of contacts sufficient to transmit infection in unit time and the probability of transmission in a contact, $\kappa$ represents the rate at which exposed individuals become infective, $\gamma$ represents the recovery rate of infective individuals, and *f* represents the disease mortality rate. *P* is the number of live individuals, $P = S + E + I + R$.

The basic reproduction number is

2 $$\begin{aligned} \mathcal {R}_0 = \frac{\beta }{\gamma }. \end{aligned}$$

The (time-dependent) effective reproduction number is

$$\begin{aligned} \mathcal {R}_t = \frac{\beta e^{-\kappa t}}{\gamma }\frac{S(t)}{P(t)}, \end{aligned}$$

and so long as the number of disease cases is small compared to the initial population size, this is approximately

$$\begin{aligned} \frac{\beta e^{-\kappa t}}{\gamma }. \end{aligned}$$

Because the model (1) is non-autonomous, there is no final size relation available, but it is not difficult to simulate the model numerically for a given set of parameter values.

## Heterogeneity in epidemic models

Another possible explanation for epidemic sizes smaller than expected is heterogeneity of mixing of individuals, and we will extend our model to epidemics which include heterogeneity.

It has been observed that in many disease outbreaks, the phenomenon of superspreading events, situations in which a small fraction of the population causes more than its share of disease cases, has been significant (Lloyd-Smith et al. 2005). For example, superspreading events were important in the SARS outbreak of 2002–2003 (Riley et al. 2003), in which there was a cluster of at least 125 cases apparently infected by a single index patient as well as another cluster of perhaps 300 cases. For a given basic reproduction number, the number of disease cases is generally fewer if there is superspreading than if the population mixing is homogeneous.

In the 2014–2015 West Africa Ebola epidemic, there is also evidence of superspreading (Lau et al. 2017). It is suggested in Galvani and May (2005) that commonly roughly 20% of the individuals in a population are responsible for 80% of the disease cases.

We have described a simple superspreading epidemic model in Brauer (2018). Here, we extend this model to include a time-dependent contact rate and disease deaths.

We consider an *SEIR* epidemic model in a population divided into two groups. Group 1 members (superspreaders), forming a fraction $\rho$ of the total population, have a contact rate $\sigma a$ and Group 2 members have a contact rate *a*, with $\sigma > 1$. Mixing between groups is proportionate. We take $\sigma = 16,\rho = 0.2$ in order to conform to the suggestion in Galvani and May (2005) that commonly roughly $20 \%$ of the individuals in a population are responsible for $80 \%$ of the disease cases.

We assume

- Total initial population size is *N*. Group 1 initial population size is $N_1 = \rho N$. Group 2 initial population size is $N_2 = (1 - \rho )N$.
- Group *i* is divided into susceptibles ($$S_i$$), exposed members ($$E_i $$), infectives ($$I_i$$), and recovered members ($$R_i$$).
- Mixing between groups is proportionate. The fraction of contacts of susceptibles with groups 1 and 2, respectively, are
  $$\begin{aligned} p_1 = \frac{\rho \sigma }{\rho \sigma + (1 - \rho )}, \quad p_2 = \frac{1 - \rho }{\rho \sigma + (1 - \rho )}. \end{aligned}$$
- Infections in each group recover at rate $\gamma$.
- There is a disease death rate *f*.
- There are no demographic effects (births, deaths, migration) on the population.

Then, the total population sizes at time *t* in the two groups are

$$\begin{aligned} P_1 = S_1 + E_1 +I_1 + R_1, \quad P_2 = S_2 + E_2 +I_2 + R_2. \end{aligned}$$

The model is (Brauer 2018)

3 $$\begin{aligned} S_1'= & {} - \sigma \beta ^* e^{- \kappa t}S_1 \left[ \frac{\rho \sigma }{\rho \sigma +(1-\rho )}\frac{I_1}{P_1} - \frac{1-\rho }{\rho \sigma + (1-\rho )}\frac{I_2}{P_2}\right] \nonumber \\ E_1'= & {} \sigma \beta ^* e^{- \kappa t}S_1 \left[ \frac{\rho \sigma }{\rho \sigma + (1-\rho )}\frac{I_1}{P_1} + \frac{1-\rho }{\rho \sigma + (1-\rho )}\frac{I_2}{P_2}\right] - \eta E_1 \nonumber \\ I_1'= & {} \eta E_1 - \gamma I_1 \\ R_1'= & {} (1 - f) \gamma I_1 \nonumber \\ S_2'= & {} - \beta ^* e^{- \kappa t}S_2\left[ \frac{\rho \sigma }{\rho \sigma + (1-\rho )}\frac{I_1}{P_1} - \frac{1-\rho }{\rho \sigma + (1-\rho )}\frac{I_2}{P_2}\right] \nonumber \\ E_2'= & {} \beta ^* e^{- \kappa t}S_2 \left[ \frac{\rho \sigma }{\rho \sigma + (1-\rho )}\frac{I_1}{P_1} + \frac{1-\rho }{\rho \sigma + (1-\rho )}\frac{I_2}{P_2}\right] - \eta E_2 \nonumber \\ I_1'= & {} \eta E_2 - \gamma I_2 \nonumber \\ R_1'= & {} (1 - f) \gamma I_2 \nonumber \end{aligned}$$

We let

$$\begin{aligned} \theta = \rho \sigma + (1 - \rho ). \end{aligned}$$

To calculate the reproduction number using the next-generation method (van den Driessche and Watmough 2002) for $t=0$ using the disease-free equilibrium with

$$\begin{aligned} P_1 = N_1 = \rho N, P_2 = N_2 = (1-\rho )N \end{aligned}$$

and

$$\begin{aligned} S_1 = \rho N, S_2 = (1-\rho )N, E-1 = E_2\,{=}\,!_1 = I_2 = R_1 = R_2 = 0, \end{aligned}$$

we write

$$\begin{aligned} F = \begin{bmatrix} 0&\quad \frac{\rho \sigma ^2 \beta ^*}{\theta }&\quad 0&\quad \frac{\sigma \rho \beta ^*}{\theta } \\ 0&\quad 0&\quad 0&\quad 0 \\ 0&\quad \frac{(1 - \rho )\sigma \beta ^*}{\theta }&\quad 0&\quad \frac{(1 - \rho )\beta ^*}{\theta } \\ 0&\quad 0&\quad 0&\quad 0 \end{bmatrix}, \quad V = \begin{bmatrix} \sigma&\quad 0&\quad 0&\quad 0 \\ - \sigma&\quad \alpha&\quad 0&\quad 0 \\ 0&\quad 0&\quad \sigma&\quad 0 \\ 0&\quad 0&\quad -\sigma&\quad \alpha \\ \end{bmatrix} \end{aligned}$$

Thus, the next-generation matrix is

$$\begin{aligned} FV^{-1} = \begin{bmatrix} \frac{\rho \sigma ^2 \beta ^*}{\alpha \theta }&\frac{\rho \sigma ^2 \beta ^*}{\alpha \theta }&\frac{\rho \sigma \beta ^*}{\alpha \theta }&\frac{\rho \sigma \beta ^*}{\alpha \theta } \\ 0&0&0&0 \\ \frac{(1 - \rho )\sigma \beta ^*}{\alpha \theta }&\frac{(1 - \rho )\sigma \beta ^*}{\alpha \theta }&\frac{\beta ^*(1 - \rho )}{\alpha \theta }&\frac{\beta ^*(1 - \rho )}{\alpha \theta } \\ 0&0&0&0 \end{bmatrix}. \end{aligned}$$

This matrix has the same nonzero eigenvalues as the $2 \times 2$ matrix

$$\begin{aligned} K_L = \begin{bmatrix} \frac{\rho \sigma ^2 \beta ^*}{\alpha \theta }&\frac{\rho \sigma \beta ^*}{\alpha \theta } \\ \frac{\beta ^*(1 - \rho )}{\alpha \theta }&\frac{\beta ^*(1 - \rho )}{\alpha \theta } \\ \end{bmatrix} \end{aligned}$$

(the next-generation matrix with least domain). The matrix $K_L$ has determinant zero. Thus, the nonzero eigenvalue is the trace of this matrix, and

$$\begin{aligned} \mathcal {R}_0 = \frac{\beta ^*}{\alpha \theta }[\sigma ^2\rho + (1 - \rho )] = \frac{\beta ^*}{\alpha } \frac{\sigma ^2 \rho + (1 - \rho )}{\sigma \rho + (1 - \rho )} \end{aligned}$$

In order to have a superspreading model with the same reproduction number as the original model (1), we must take

$$\begin{aligned} \beta ^* = \beta \frac{\theta }{\sigma ^2 \rho + (1 - \rho )}. \end{aligned}$$

## Guinea: An Example

The first country to experience an outbreak of Ebola in 2014 was Guinea, where the index case occurred on December 2, 2013. The development of the disease in Guinea was modeled by (1) with parameter values

$$\begin{aligned} N = 10{,}589{,}000, \quad \beta = 0.27, \quad \eta = 1/5.3, \quad \gamma = 1/5.61, \quad \kappa = 0.0023, f = 0.71, \end{aligned}$$

giving a basic reproduction number $\mathcal {R}_0 = 1.51$. In fact, there were 3814 cases of disease in Guinea.

The model (1) was simulated using Maple, with initial values

$$\begin{aligned} S(0) = N - 40, \quad E(0) = 0, \quad I(0) = 40, \quad R(0) = 0. \end{aligned}$$

If $\kappa = 0$, the situation in which there is no decrease in the basic reproduction number, we would obtain $S_\infty = 1{,}182{,}015$, corresponding to 9, 406, 985 disease cases (including the initial infectives). With the given estimate of $\kappa$, we would obtain $S_\infty = 10{,}561{,}299$, corresponding to 27, 701 disease cases, much closer to the actual number of cases.

Simulation, using Maple, of the superspreader model (3), with $S_1(0) = 2{,}117{,}792, S_2(0) = 8{,}491{,}168$, lead to $S_1(\infty ) = 2{,}099{,}272, S_2(\infty ) = 8{,}486{,}508$ corresponding to 23, 220 disease cases. a slightly better estimate.

## Sierra Leone: An Example

The first cases of Ebola in Sierra Leone were reported on May 27, 2014. The development of the disease in Guinea was modeled by (1) with parameter values

$$\begin{aligned} N = 5{,}364{,}000, \beta = 0.45, \eta = 1/5.3, \gamma = 1/5.61, \kappa = 0.0097, f = 0.48, \end{aligned}$$

giving a basic reproduction number $\mathcal {R}_0 = 2.53$. In fact, there were 14,124 cases of disease in Guinea.

The model (1) was simulated using Maple, with initial values

$$\begin{aligned} S(0) = N - 20, \quad E(0) = 0, \quad I(0) = 20, \quad R(0) = 0. \end{aligned}$$

If $\kappa = 0$, the situation in which there is no decrease in the basic reproduction number, we would obtain $S_\infty = 206{,}896$, corresponding to 5, 157, 104 disease cases (including the initial infectives). With the given estimate of $\kappa$, we would obtain $S_\infty = 5{,}341{,}699$, corresponding to 22, 301 disease cases, much closer to the actual number of cases.

Simulation, using Maple, of the superspreader model (3), with $S_1(0) = 106{,}919, S_2(0) = 4{,}276{,}784$, leads to $S_1(\infty ) =1{,}058{,}093, S_2(\infty ) = 4{,}273{,}995$ corresponding to 13, 912 disease cases. a slightly better estimate.

## Liberia: An Example

The Ebola epidemic in Liberia began somewhat later than the epidemics in Guinea and Sierra Leone. There was a minor outbreak in April 2014, and the first cases in the full epidemic were reported on June 16, 2014. Fisman et al. (2014) found no decrease in the reproduction number by early September 2014. The data after early September are incomplete and unreliable, and the only available estimate of a decrease in the reproduction number for Ebola in Liberia is in WHO Ebola Response Team (2014) which leads to a decrease rate of $\kappa = 0.0018$, probably smaller than the actual rate.

Simulation of the model (1) using Maple was carried out, with parameter values

$$\begin{aligned} N = 3{,}787{,}000, \beta = 0.28, \eta = 1/5.3, \gamma = 1/5.61, \kappa = 0.0018, f = 0.71, \end{aligned}$$

and initial values

$$\begin{aligned} S(0) = N - 20, \quad E(0) = 0, \quad I(0) = 20, \quad R(0) = 0. \end{aligned}$$

If $\kappa = 0$, the situation in which there is no decrease in the basic reproduction number, we would obtain $S_\infty = 585{,}843$, corresponding to 3, 701, 157 disease cases (including the initial infectives). With the given estimate of $\kappa$, we would obtain $S_\infty = 3{,}683{,}495$, corresponding to 103, 505 disease cases, much closer to the actual number of cases but still quite large.

Simulation, using Maple, of the superspreader model (3), with $S_1(0) = 757{,}396, S_2(0) = 3{,}029{,}584$, leads to $S_1(\infty ) =724{,}293, S_2(\infty ) = 3{,}021{,}121$ corresponding to 41, 636 disease cases, a considerably better estimate.

## Conclusions

In order to plan management strategies for an outbreak of an epidemic, it is important to have an estimate early in the epidemic of how serious the epidemic will be. For an epidemic that may spread very widely, an estimate based on a simple *SIR* or *SEIR* model may predict a very large number of disease cases. If the disease is viewed as very dangerous, there may be substantial behavioral changes in the affected population even before official control measures are begun. These may lead to a decrease in the reproduction number of the epidemic and a much smaller number of disease cases than an initial prediction. We suggest that it would be very useful to estimate both the (initial) basic reproduction number and an effective reproduction number a little later in the epidemic in order to estimate how the reproduction number changes over the course of the epidemic. The estimate may be improved further by assuming heterogeneity of mixing, which appears to be an extremely common phenomenon. Reasonably accurate initial estimates are useful in planning the overall management of an epidemic, which may be quite distinct from the problems of management in individual regions.

There is a possible contradiction in this procedure. Behavioral response to an epidemic may be much larger if the public estimates of epidemic size are estimates that do not assume behavioral response. It would be important to emphasize how large the effect of even small decreases in reproduction number can be on the size of an epidemic.
